# Critical appraisal of RapidArc radiosurgery with flattening filter free photon beams for benign brain lesions in comparison to GammaKnife: a treatment planning study

**DOI:** 10.1186/1748-717X-9-119

**Published:** 2014-05-21

**Authors:** Ufuk Abacioglu, Zeynep Ozen, Meltem Yilmaz, Alptekin Arifoglu, Basri Gunhan, Namik Kayalilar, Selcuk Peker, Meric Sengoz, Salih Gurdalli, Luca Cozzi

**Affiliations:** 1 Neolife Medical Center, Istanbul, Turkey; 2 Acibadem Kozyatagi Hospital GK Center, Istanbul, Turkey; 3 Medical Physics Unit, Oncology Institute of Southern Switzerland, 6504 Bellinzona, Switzerland

**Keywords:** SRS, RapidArc, GammaKnife, Schwannoma, Meningioma

## Abstract

**Background:**

To evaluate the role of RapidArc (RA) for stereotactic radiosurgery (SRS) of benign brain lesions in comparison to GammaKnife (GK) based technique.

**Methods:**

Twelve patients with vestibular schwannoma (VS, n = 6) or cavernous sinus meningioma (CSM, n = 6) were planned for both SRS using volumetric modulated arc therapy (VMAT) by RA. 104 MV flattening filter free photon beams with a maximum dose rate of 2400 MU/min were selected. Data were compared against plans optimised for GK. A single dose of 12.5 Gy was prescribed. The primary objective was to assess treatment plan quality. Secondary aim was to appraise treatment efficiency.

**Results:**

For VS, comparing best GK vs. RA plans, homogeneity was 51.7 ± 3.5 vs. 6.4 ± 1.5%; Paddick conformity Index (PCI) resulted 0.81 ± 0.03 vs. 0.84 ± 0.04. Gradient index (PGI) was 2.7 ± 0.2 vs. 3.8 ± 0.6. Mean target dose was 17.1 ± 0.9 vs. 12.9 ± 0.1 Gy. For the brain stem, D_1cm3_ was 5.1 ± 2.0 Gy vs 4.8 ± 1.6 Gy. For the ipsilateral cochlea, D_0.1cm3_ was 1.7 ± 1.0 Gy vs. 1.8 ± 0.5 Gy. For CSM, homogeneity was 52.3 ± 2.4 vs. 12.4 ± 0.6; PCI: 0.86 ± 0.05 vs. 0.88 ± 0.05; PGI: 2.6 ± 0.1 vs. 3.8 ± 0.5; D_1cm3_ to brain stem was 5.4 ± 2.8 Gy vs. 5.2 ± 2.8 Gy; D_0.1cm3_ to ipsi-lateral optic nerve was 4.2 ± 2.1 vs. 2.1 ± 1.5 Gy; D_0.1cm3_ to optic chiasm was 5.9 ± 3.1 vs. 4.5 ± 2.1 Gy. Treatment time was 53.7 ± 5.8 (64.9 ± 24.3) minutes for GK and 4.8 ± 1.3 (5.0 ± 0.7) minutes for RA for schwannomas (meningiomas).

**Conclusions:**

SRS with RA and FFF beams revealed to be adequate and comparable to GK in terms of target coverage, homogeneity, organs at risk sparing with some gain in terms of treatment efficiency.

## Background

Vestibular Schwannoma (VS) is a benign tumor that originates from the vestibular portion of the eighth cranial nerve. The incidence is 1 in 100,000. The patients due to the mass effect may note symptoms like disequilibrium, vertigo, tinnitus and headache. However, most patients complain from progressive unilateral hearing decline. Earlier detection with widespread use of MR imaging prompts the patient and the clinician to treatment, when the patient still has useful hearing. Management options are observation, microsurgery, stereotactic radiosurgery (SRS) or fractionated stereotactic radiotherapy.

Intracranial meningiomas are mostly benign tumors (WHO grade I) in 90-95% of the cases. Clinical behavior and treatment strategies depend on the location, size and proximity to critical structures. Surgical treatment is preferred for easily accessible tumors and gross total resection achieves >90% local control rates. However, surgery for the meningiomas in the skull base like cavernous sinus meningiomas (CSM) carries high morbidity risk. SRS for CSM with maximum diameter less than 3 to 4 cm and with some distance from critical structures provides high local control rates with reasonable morbidity [1].

The stereotactic radiosurgery (SRS) approach to the treatment of small to medium sized VS or meningioma is well consolidated in literature with results demonstrating both very high local control rates (larger than 90% up to 96-98%) as well as minimal toxicity (5 yr radiation related toxicity as low as ~5-10%) [2-7]. Recent data suggest that good control rates and low side effects can be achieved with doses around 12-13 Gy [7].

For VS, the rate of post-treatment hearing preservation is reported to be strongly correlated with the radiation dose to the cochlea. Mean cochlea doses below 3.7 and 4.8 Gy are reported to be safe for retaining useful hearing [8]. QUANTEC guideline recommends maximum dose to be limited to 12-14 Gy for hearing preservation [9]. For the CSM, the highest risk of toxicity in SRS, might derive from radiation induced optic neuropathy. QUANTEC [10] identified that with near-to-maximum doses <8 Gy, the risk should be rare.

Several different techniques have been applied in the last decades; these were based either on the usage of dedicated delivery systems or on general purpose linear accelerators, modified or adapted to SRS.

Dedicated systems included mainly the GammaKnife (GK) originally developed by Leksell [11] and more recently the CyberKnife, derived from the intuition of Adler [12].

Sun [13] demonstrated on a group of 200 patients that a 5 year control rate of 93% was expected with a 92% hearing preservation for VS patients treated with <14 Gy and GK. Similar results were reported by Hasegawa [7] on a cohort of 400 patients followed for more than 10 years. Zeiler [14] showed efficacy of GK based radiosurgery of CSM on a cohort of 30 patients. Balagamwale [15] showed, on 145 patients with meningiomas, the role of various dose-related indices suggesting the need of high conformality of treatments. Starke [16] analysed retrospectively 225 patients with skull base meningioma, of these 146 received GK treatment after surgery. This study showed actuarial progression free survival rate of 96% at 5 years (79% at 10 years).

Based on general purpose linear accelerators, common techniques included the usage of multiple conformal arcs (with cones or micro-MLC) or multiple static intensity modulated (IMRT) fields. Today, IMRT evolved into volumetric modulated arc therapy (VMAT) pioneered in its RapidArc (RA) mode by Otto [17]. The role of RA was assessed for SRS for benign lesions in a number of studies. Fogliata [18] demonstrated the possibility to achieve target coverage equivalent to Helical Tomotherapy in the presence of improved sparing of organs at risk. Lagerwaard [19] approached the usage of RA for VS demonstrating increased conformality and advanced dose shaping potential compared to conformal arcs. Subramanian [20] investigated the role of RA on (large) AVMs and reported well-tolerated treatments with minimal toxicity. More in general, Mayo [21], Clark [22] and Wolff [23] analysed the general role of RA for SRS and demonstrated relevant improvements in plan quality and also in delivery efficiency when compared to alternative techniques (e.g. vs. CyberKnife in the study of Mayo). A relevant aspect of RA based SRS is the possibility to accurately plan with the existing tools. Audet [24] demonstrated the absence of lower limits in the size of lesions for which RA plans can be optimized. Fogliata [25] demonstrated that treatment planning and dose calculation tools can be properly tailored for SRS planning and calculated dose can be nicely matched to delivery.

The purpose of the present study is to assess the potential differences and relative merits of the RA based radiosurgery in comparison to the GK approach. The primary aim is to quantify dose-related objective differences of clinical relevance. The secondary aim of the study is to determine the delivery efficiency of the two methods in terms of treatment time (not including positioning and imaging procedures which are very different and not comparable between the systems).

## Materials and methods

### Patients, volumes and dose prescriptions

Six VS cases who were treated with RA technique and 6 CSM cases who were treated by GK formed the basis of this study. They were randomly selected from the pool of previously treated patients. Median age was 40 years (range, 15–63) for VS and 64 years (range, 57–66) for CSM. Male/female ratio was 5/1 for VS and 2/4 for CSM. VS and CSM cases had mean tumor volumes of 4.2 cc (range, 1.2-8.5 cc) and 7.9 cc (range, 3.3-13.7 cc), respectively. They represented the range of target sizes and shapes mostly observed in SRS practice.

CT scans with contrast were acquired with 1.3 mm slice thickness covering the entire head of the patients. MR image acquisition was performed with head coil on a 1.5 or 3 Tesla MR. T1 sequences with gadolinium was used for Gross Target Volume (GTV) delineation and CISS sequence was used for internal auditory structures. All images were co-registered for target volume and organ at risk (OAR) delineation. GTV definition was performed by experienced radiation oncologists and neurosurgeons based on appropriate multimodality imaging (CT and MR). No margins were added to the GTV to define the Planning Target Volume (PTV). There is no absolute consensus about the need to add a little margin to GTV when non invasive immobilisation is used [26] but in this study, to perform an unbiased comparison, this was not adopted. OARs were accordingly defined by the same team. Data transfer between different systems was performed by means of DICOM and the same structures were used for both GK and RA planning.

The study was performed in agreement with the Helsinki declaration and with internal institutional ethical review board approval.

For the VS patients, the brain stem and the ipsi-lateral cochlea were defined as the organs at risk. For the CSM patients, the OAR were the brain stem, the ipsi-lateral optic nerve and the chiasm.

### Dose prescription and planning objectives

A single fraction dose of 12.5 Gy was prescribed to all cases. All plans were normalised to guarantee the same minimum coverage to GTV so that V_98_ = 98% of the prescribed dose. A common normalisation is mandatory for any comparative purpose. Planning objectives were kept as simple as possible and identical between the different systems: minimisation of mean and near-to-maximum doses to the organs at risk without compromising target coverage. Observed differences between dose distributions would therefore reflect the inherent features of those and allow a more informed choice between different solutions. To further explore technical capabilities of linac based SRS, RA plans were optimised with two strategies: TB_1: enforcing minimisation of the homogeneity, that means minimisation of the near-to-maximum dose D2%. The second strategy, TB_2, aimed to mimic the GK planning philosophy with no upper constraints to the GTV dose. TB_1 plans have been considered to investigate if RA and FFF could guarantee adequate target coverage and OARs sparing while eliminating the target over-irradiation which could constitute a change of paradigm in the SRS planning practice. Similarly to the above, GK_1 plans were obtained by applying strong priority to the maximisation of the dose gradient out of the target while GK_2 aimed also to exploit maximal OAR sparing.

### SRS with RapidArc

For all patients in the study, RA plans were optimised using 4–8 partial non-coplanar arcs. Collimator and couch pedestal angles as well as single arc lengths were optimised for each individual case with trial and error procedure. Inverse planning was performed with the Varian Eclipse treatment planning system (version 10) and dose calculation with the Acuros-XB algorithm with a grid resolution of 1 mm. All plans were designed for a 10 MV flattening filter free beam generated by a TrueBeam (TB) linac equipped with a high definition MLC (120 leaves with a spatial resolution at isocenter of 2.5 mm in the region of interest for the study). To improve healthy tissue sparing, the jaw tracking option of TB was enabled for all patients. Similar dosimetric results could be achievable with the lower 6 MV FFF beam but the choice of 10 MV was made to benefit from the higher nominal dose rate (2400 MU/min versus 1400 MU/min).

### SRS with GammaKnife

GK plans were optimised for all patients using the Elekta GammaPlan treatment planning system (version 10.1) for a GK Perfexion treatment unit. The GK has 192 Co-60 sources, which are placed on 8 sectors. Each sector can move in a linear direction back and forth over the internal collimation system with several stopping positions. Each position corresponds to a different size collimator. Each sector has 24 sources, 3 different size of open collimators are available for each source (16 mm, 8 mm, 4 mm) as well as a blocked collimator. Therefore there is no more requirements to manually change collimation helmets as with earlier versions of GK. All sources are used by default. However to protect the organs at risk in the neighborhood of the target, the optimization process allows to block one or more sectors or even individual sources. Because each of the 8 sectors can move independently, it is possible to create plans with composite multiple isocentres (shots) where each sector is of different collimator size. This can be done with manual trial and error procedure or with inverse planning (as in the present study): the algorithm can automatically block the sectors/sources, which come through the OAR. Concerning GK_1 plans, for the VS cases, an average of 26 ± 8 shots were used (range 14–36) with 8 mm collimator in 35% and 4 mm in 57% of the cases (only 1 shot for a single patient was associated to a collimator of 16 mm). For CSM cases, 25 ± 7 shots were used (range:18–32) with 4 mm in 51% of cases, 8 mm in 45% and 16 mm in 4% of the total shots. Concerning GK_2 plans, for the VS cases and average of 14 ± 4 shots (range 10–21) were used with a proportion of collimator sizes similar to GK_1. For the CSM cases the number of shots was 20 ± 6 (range: 15–32),

Dose calculation was performed with the TMR classic algorithm. Calculation matrix was set to 2.5 mm for all plans. All dose data were exported in DICOM format and imported in Eclipse for quantitative analysis (eliminating therefore one important bias risk from the usage of different methods for DVH calculation).

### Analysis tools

For each patient, dosimetric parameters were scored and plan quality was measured from dose volume histogram (DVH) analysis. For GTV, target coverage (minimum as D_98%_, maximum as D_2%_) was reported. Homogeneity was scored as (V_5%_-V_95%_)/D_mean_ as well as in terms of standard deviation. Paddick Conformity and Gradient indexes (PCI and PGI) were defined and reported [27,28]. PCI = TV_PIV_^2^/(TV x PIV) where TV is the target volume, TV_PIV_ is the target volume irradiated at prescription dose and PIV is the prescribed isodose volume; PGI = V_50%PIV_/PIV where V_50%PIV_ is the volume irradiated at 50% of the prescribed dose. In addition, a Dose Gradient Measure (DGM) was defined as the difference of the radii of the equivalent spheres of the 100% and 50% isodose volumes. For OARs, the mean dose, the maximum dose (D_xcm3_) and appropriate values of V_xGy_ (volume receiving at least x Gy) were scored depending on the individual organ. Guidelines of International Commission on Radiation Units and Measurements (ICRU) 83 report were applied as much as possible [29]. No analysis on the “body” healthy tissue was performed since the dose matrices of the GK did not covered the entire volume involved.

Treatment time for RA plans was measured at the linac while for GK plans was computed by the planning system. The assessment also did not accounted for door-to-door time estimate since this might depend upon several variables (local procedures, resources, institutional policies) that might be irreproducible between different centers. We preferred to limit the analysis to objective quantities, independent from the local boundary conditions. This assessment did not included pre-treatment quality assurance verification since this is part of the planning acceptance procedure and depends significantly on the methodology used. Both systems (GK and TB with RA) are in clinical use (also for SRS) since long time and several publications addressed the plan deliverability issue in detail.

## Results

**Figure 1:**
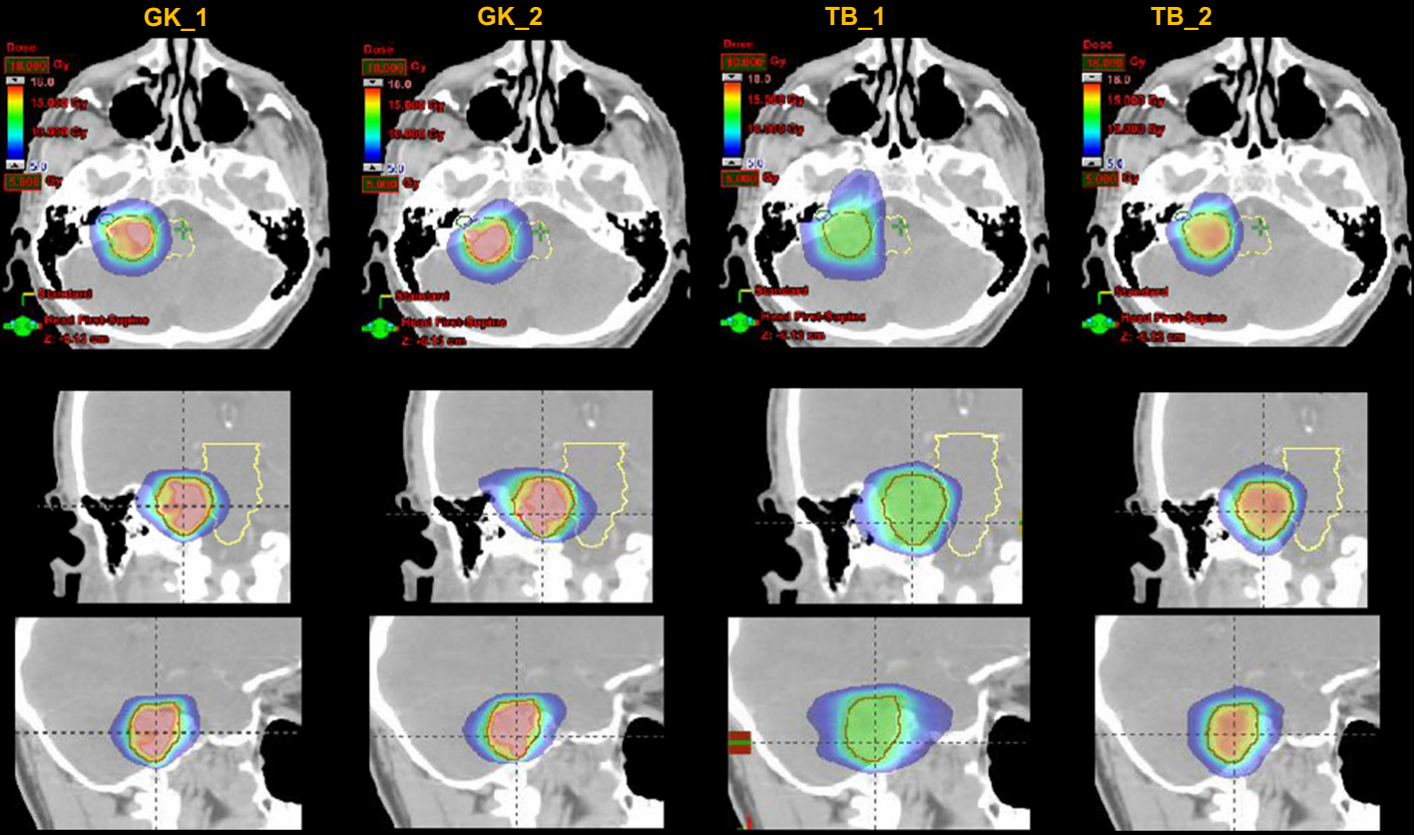
**The isodose distribution for the four plans in axial, coronal and sagittal planes for a representative Schwannoma case.** Colorwash threshold is set between 5 and 18Gy.

**Figure 2:**
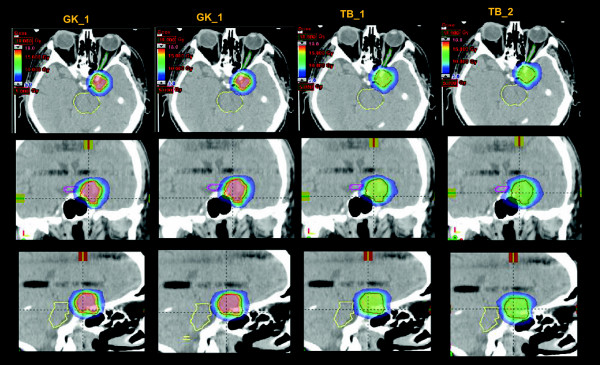
**The isodose distribution for the four plans in axial, coronal and sagittal planes for a representative Meningioma case.** Colorwash threshold is set between 5 and 18 Gy.

Figure 1 shows the isodose distribution for the four plans in axial, coronal and sagittal planes for a representative VS case. Figure 2 shows the same for a CSM case. Color-wash threshold was set between 5 and 18 Gy. From the figures, it is possible to derive the qualitative features of the plans: equivalent coverage between all techniques (required at planning); intentionally homogeneous dose distributions for TB_1 plans and systematic over-irradiation of target, as from conventional practice with SRS, for GK_1, GK_2 and TB_2.

**Figure 3:**
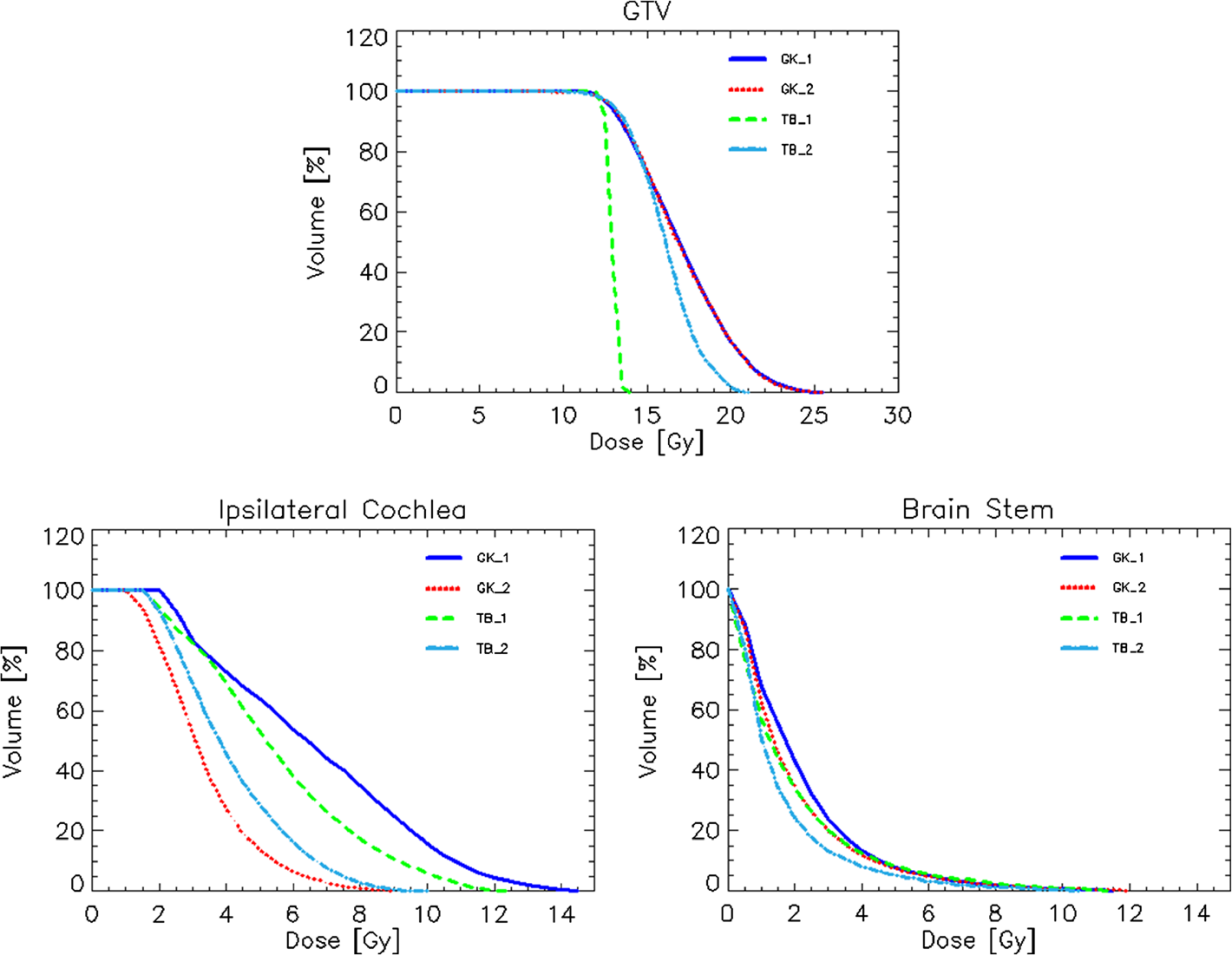
Average DVH for target volume and organs at risk for the schwannoma patients and the four techniques.

**Figure 4:**
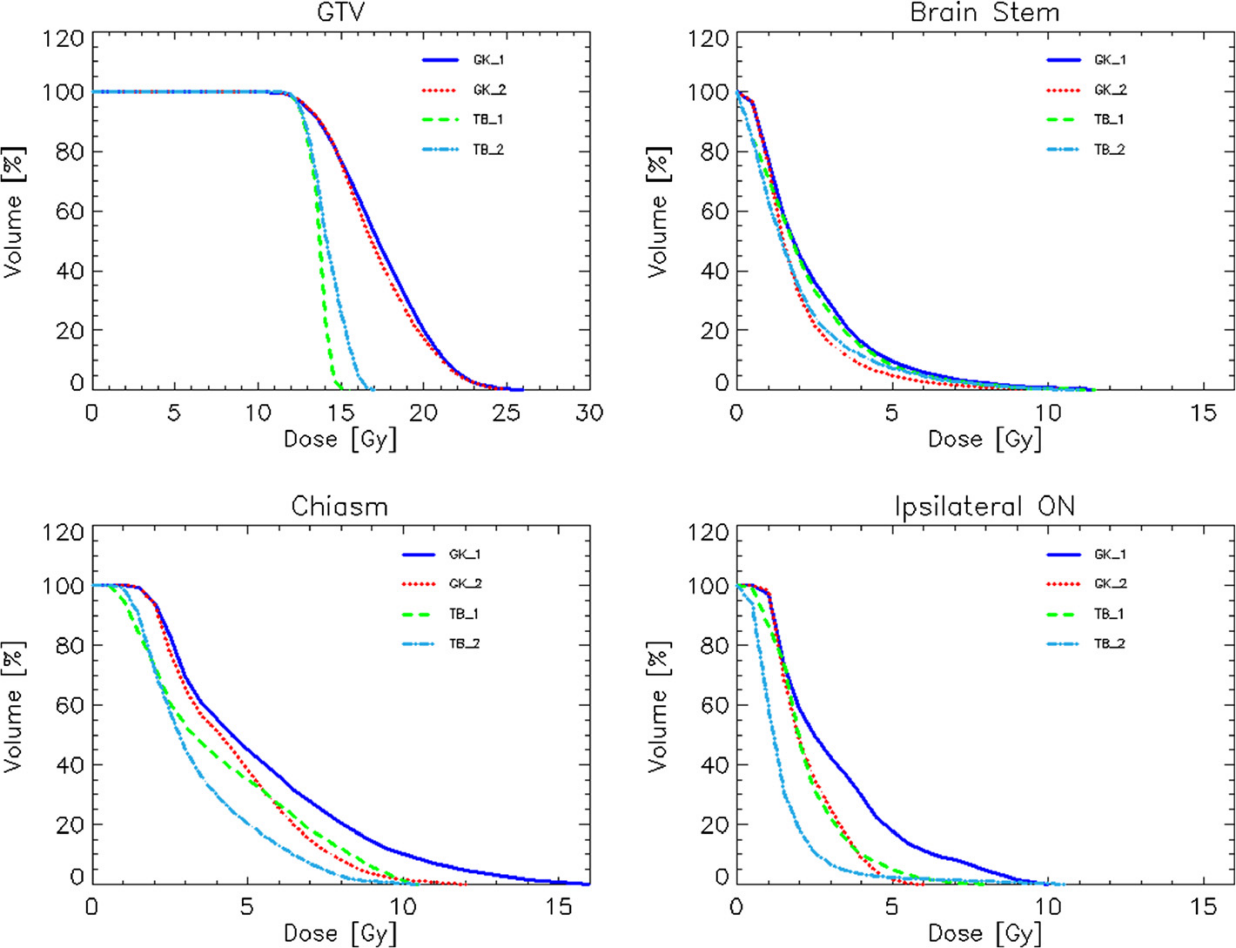
Average DVH for target volume and organs at risk for the meningioma patients and the four techniques.

Figure 3 shows the average DVH for target volume and organs at risk for the VS cases and the four techniques while Figure 4 shows the same for the CSM group.

Table 1 summarises the quantitative analysis of the DVH for the VS cases for GTV and organs at risk. Table 2 presents the same for the CSM cases.

**Table 1:** Summary of dose volume histogram analysis for GTV, brain stem and ipsilateral cochlea for VS patients

	**GK_1**	**GK_2**	**TB_1**	**TB_2**	**p**
	**PTV vol [cm**^**3**^**]: 4.22 ± 2.85 Range: [1.20; 8.50]**				
Mean [Gy]	17.1 ± 0.9	17.1 ± 0.8	12.9 ± 0.1	16.0 ± 0.6	a, b, c, d
D_2%_ [Gy]	22.7 ± 1.2	23.0 ± 0.8	13.4 ± 0.2	18.8 ± 1.1	a, b, c, d
D_98%_ [Gy]	12.25 ± 0.0	12.25 ± 0.0	12.25 ± 0.0	12.25 ± 0.0	n.s.
St. dev [Gy]	2.7 ± 0.3	2.8 ± 0.2	0.3 ± 0.1	1.7 ± 0.3	a, b, c, d
Homogeneity [%]	51.7 ± 3.5	51.8 ± 1.2	6.4 ± 1.5	35.0 ± 4.9	a, b, c, d
PCI	0.81 ± 0.03	0.78 ± 0.04	0.83 ± 0.07	0.84 ± 0.04	c, d
PGI	2.7 ± 0.2	2.8 ± 0.2	5.1 ± 0.7	3.8 ± 0.6	a, b, c, d
DGM [cm]	0.4 ± 0.1	0.4 ± 0.1	0.6 ± 0.1	0.5 ± 0.1	a, b, c, d
	**Brain stem vol [cm**^**3**^**] = 21.50 ± 8.37 Range: [8.69; 32.70]**				
D_1cm3_ [Gy]	5.1 ± 2.0	5.4 ± 2.2	5.7 ± 2.1	4.8 ± 1.6	n.s.
V_8Gy_ [%]	1.9 ± 1.7	2.3 ± 1.8	2.5 ± 1.9	1.1 ± 1.0	a, b, d
V_10Gy_ [%]	0.6 ± 0.5	0.8 ± 0.6	0.8 ± 0.7	0.2 ± 0.2	a, b, d
V_12Gy_ [%]	0.1 ± 0.1	0.2 ± 0.2	0.03 ± 0.04	0.01 ± 0.01	a
	**Ipsilateral cochlea vol [cm**^**3**^**] = 0.10 ± 0.01 Range: [0.02;0.13]**				
Mean [Gy]	6.6 ± 2.9	3.3 ± 0.8	5.5 ± 2.2	4.1 ± 0.9	a, b, c
D_0.1cm3_ [Gy]	4.0 ± 1.8	1.7 ± 1.0	3.1 ± 1.2	1.8 ± 0.5	a, b, c
D_30%_ [Gy]	7.3 ± 3.2	3.6 ± 0.8	6.2 ± 2.4	4.7 ± 1.1	a, b, c, d

Statistical significance p: a = GK_1 vs TB_1, b = GKv vs TB_2, c = GK_2 vs TB_1, d = GK_2 vs TV_2; n.s. = not significant. PCI = Paddick Conformity Index, PGI = Paddick Gradient Index, DGM = Dose Gradient measure. Dx% = Dose at x% of the volume.

**Table 2:** Summary of dose volume histogram analysis for GTV, brain stem, optic chiasm and ipsi-lateral optic nerve for CSM patients

	**GK_1**	**GK_2**	**TB_1**	**TB_2**	**p**
	**PTV vol [cm**^**3**^**]: 7.28 ± 4.2 Range: [3.3; 13.7]**				
Mean [Gy]	17.4 ± 0.9	17.4 ± 0.9	13.6 ± 0.2	14.2 ± 0.5	a, b, c, d
D_2%_ [Gy]	22.9 ± 1.4	23.2 ± 1.3	14.4 ± 0.4	15.8 ± 0.8	a, b, c, d
D_98%_ [Gy]	12.25 ± 0.0	12.25 ± 0.0	12.25 ± 0.0	12.25 ± 0.0	-
St. dev [Gy]	2.8 ± 0.2	2.8 ± 0.2	0.5 ± 0.1	0.9 ± 0.3	a, b, c, d
Homogeneity [%]	52.3 ± 2.4	52.7 ± 1.8	12.4 ± 0.6	21.1 ± 0.5	a, b, c, d
PCI	0.86 ± 0.05	0.81 ± 0.06	0.86 ± 0.06	0.88 ± 0.05	c, d
PGI	2.7 ± 0.1	2.6 ± 0.1	3.9 ± 0.7	3.8 ± 0.5	a, b, c, d
DGM [cm]	0.5 ± 0.1	0.5 ± 0.1	0.7 ± 0.1	0.6 ± 0.1	a, b, c, d
	**Brain stem vol [cm**^**3**^**] = 19.9 ± 4.3 Range: [15.36; 26.1]**				
D_1cm3_ [Gy]	5.4 ± 2.8	5.5 ± 2.6	5.8 ± 2.0	5.2 ± 2.8	n.s.
V_8Gy_ [%]	2.3 ± 2.7	2.3 ± 2.6	1.8 ± 1.7	1.8 ± 1.6	n.s.
V_10Gy_ [%]	0.7 ± 0.8	0.7 ± 0.7	0.5 ± 0.6	0.4 ± 0.4	n.s.
V_12Gy_ [%]	0.1 ± 0.1	0.1 ± 0.1	0.1 ± 0.1	0.0 ± 0.1	n.s.
	**Optic chiasm vol [cm**^**3**^**] = 0.5 ± 0.3 Range: [0.3; 0.9]**				
D_0.1cm3_ [Gy]	6.8 ± 3.4	5.9 ± 3.1	5.1 ± 2.9	4.5 ± 2.1	a, b, d
	**Ipsilateral optic nerve vol [cm**^**3**^**] = 0.5 ± 0.1 Range: [0.4;0.5]**				
D_0.1cm3_ [Gy]	4.2 ± 2.1	4.2 ± 2.1	2.9 ± 1.6	2.1 ± 1.5	a, b, c, d

Statistical significance p: a = GK_1 vs TB_1, b = GK_1 vs TB_2, c = GK_2 vs TB_1, d = GK_2 vs TB_2; n.s. = not significant. PCI = Paddick Conformity Index, PGI = Paddick Gradient Index, DGM = Dose Gradient measure. Dx% = Dose at x% of the volume.

From a qualitative point of view, there are some general features of GK and RA plans. In the absence of upper constraints, GK generates a much more intense over-irradiation of the target, also compared to TB_2. On the contrary, RA plans (both TB_1 and TB_2) can shape more the dose around challenging OARs compared to the simpler GK_1 planning approach. GK_2 plans allowed further sparing of OARs without compromising other parameters.

The chosen normalisation to V_98_ = 98% to guarantee the same coverage for all techniques, reflected into some little variability in the minimum dose level encompassing the entire target. This resulted 70% for all VS plans and between 80 and 85% for all CSM plans. Conformality (PCI) resulted equivalent between all the groups of plans while significant difference were observed in other parameters. More homogeneous dose distributions to the target were achieved with RA (intentionally for TB_1 but also for TB_2, depending on the clinical case) if needed or desired. In the case of VS the gain in homogeneity was ~17% for TB_2 and ~45% for TB_1 compared to GK plans. For CSM the gains were ~31% for TB_2 and 40% for TB_1. This effect derived from the corresponding reduction of the near-to-maximum dose to GTV (~4 Gy (18%) sparing for TB_2 and ~9 Gy (41%) for TB_1 for VS and ~8.5 Gy and 7 Gy for CSM). The trade-off was an increase of gradients (DGM) that was observed for RA although this was limited to 1 mm in the case of GK vs. TB_2 (similarly reflected in the PGI). This trade-off in the gradients was associated to a (frequently) statistically significant improved sparing of the organs at risk. In the case of VS, the results obtained for the ipsi-lateral cochlea revealed that, depending on the optimisation strategy applied, both GK and TB plans can reduce the mean and near-to-maximum doses to very low values. Interestingly, GK plans showed the greatest improvement in sparing this OAR (GK_1 vs GK_2) when explicit constraints were applied or not. Mean dose with GK_2 and TB_2 resulted to be compatible with the range of tolerable levels for useful hearing retention. In the case of meningiomas, the near-to-maximum dose to optic chiasm was reduced of ~34% (25%) for TB_2 (TB_1) vs GK_1 and of ~24% (~15%) vs GK_2. The same parameter for the ipsi-lateral optic nerve was reduced by a factor 2 with TB_2 (31% for TB_1) vs. GK_1 or GK_2.

Treatment efficiency was scored by the treatment time (defined excluding the time needed for patient positioning and imaging procedures). This parameter ranged from 53 to 84 minutes for GK plans (with GK_2 plans requiring 6–20 minutes less than GK_1). Treatment time was consistently ~5 minutes for all TB cases.

## Discussion

Role of SRS in the treatment of intracranial benign tumors has been well established over years. Various techniques using frame-based or frameless positioning and localisation systems have proved their success. Both dedicated and general purpose radiotherapy equipments with special SRS related improvements have been developed. Comparisons of dosimetric quality and clinical results have always been an ongoing subject of investigation.

The usage of RA with flattening filter free beams was investigated in this study for the delivery of SRS treatments to VS and CSM. RA plans were compared versus GK plans and dose-related analysis was performed. In general, plans of high quality were obtained for all techniques but some general features were outlined. A limit of any planning study is the difficulty to factorise between results inherent to the techniques investigated or imputable to the choices made by the actual planners using the tools. The present study was done aiming to achieve the best plans from both systems and, for the more investigational group of RA, efforts were made to understand the degree of flexibility of the technique for three important features: dose gradients, target heterogeneity and OAR sparing. In general, GK plans confirmed the inherent capability of the technique to emphasize conformality and rapid isotropic dose fall-off associated to a good OAR sparing if required and at the price of a greater target heterogeneity. RA demonstrated a significant flexibility in the trade-off between target homogeneity and OAR sparing. The price paid was an inferior isotropic falloff and a shallower gradient. Clinical data would be now needed to demonstrate if the modest differences in dose gradient and patterns of OAR sparing could also lead to reduced side effects. The data from the present study suggest, at least, that treatment plan individualisation can be achieved with both RA and GK. Target coverage was defined equal between techniques by definition; for this reason, a further measure of the plan quality was given by PCI and various gradient measures. RA and GK resulted in equivalent conformality while plans from GK showed sharper dose gradients.

Although common practice in SRS emphasizes high conformality and sharp dose fall-off over target dose homogeneity, there is no absolute evidence or consensus that improved target homogeneity would be detrimental for disease control. An interesting and not intuitive correlation between homogeneity and gradient indexes and treatment related toxicity was observed and reported by Balagamwala [15]. In that study, patients with PGI larger than 3.0 were associated with lower incidence of motor or auditory deficits compared to patients treated with sharper dose distributions. Under these assumptions, the TB_1 and TB_2 plans would fit in the favourable category. The same study correlated also homogeneity to toxicity (dizziness) finding that a maximum dose <2 times the prescription (heterogeneity index) dose would have been recommended. Data from the present study suggest therefore that all techniques (GK or RA) would meet the criterion. The flexibility of RA in minimising the D_2%_ and the homogeneity indices (HI or standard deviation) could be considered as an additional tool available whenever the clinical problem would demand for it and, in any case, would not necessarily be always beneficial but at the same time it should not be negative.

A potential criticism to the data presentation of this study could arise from the dose normalisation chosen for the comparison. All plans were normalised so that V_98_ = 98% of the prescribed dose. This procedure, might look unusual in SRS practice but it is an obvious mandatory step in any comparative investigation to give relative meaning to any observed difference between the techniques studied. Conversely, the normalisation choice did not introduce any violation of the main SRS prescription paradigm that conventionally requires that the dose should be prescribed to the highest isodose encompassing the target volume (D_100%_) and that this should fall in the range 50-90%. In the present study, D_100%_ ranged from 80 to 85% for CSM and was 70% for the VS cases and for all techniques.

The secondary end point of the study was the assessment of the treatment efficiency. In this case, the data reported demonstrate that, compared to a treatment time of ~1 hour for GK, the same delivery can be completed in less than 5 minutes with RA. Strength of the GK sources can obviously compensate only partially this time difference. On the contrary, the benefit of the usage of flattening filter free beams with the very high nominal dose rate of 2400 MU/minute is mitigated in this study by the number of partial arcs selected for the plans. The latter was in the range of 4–8 in order to guarantee the desired high quality of dose distributions but does not necessarily correspond to an absolute best selection. Further geometrical optimisation of RA plans might lead to reduced number of arcs and to further reduction of the expected delivery time. In the extreme case of a single full arc, with the prescription of 12.5 Gy and for a beam of 10 MV FFF, the minimum beam on time would have been 1.9 minutes and this would constitute the lower limit for this case of RA application. This analysis is also consistent with the data by Prendergast [30] who reported an average treatment time of ~1.2 minutes for a variety of dose fractions ranging from 5 Gy to 16 Gy. With the data presented here, it is obvious that SRS treatments can be easily fit into routine time slots if RA is applied and that, as a consequence, minimal perturbation of the standard clinical workflow and high patient throughput can be granted also in clinical environments with relevant incidence of SRS treatments.

## Conclusion

The present study assessed in silico at planning level, the differences and relative merits of the RA based radiosurgery in comparison to the GK approach. Both approaches resulted in fully acceptable plans with different dosimetric characteristics. A possibly increased homogeneity of target dose with linac based SRS was associated to a modest deterioration of the dose gradient. Depending on the optimisation strategy, both GK and TB achieved high sparing of OARs. The clinical relevance of these features should be proven in a prospective setting. Similarly, the results for treatment time demonstrated the major gain potentially offered by RA SRS for the clinical throughput and the workflow logistic in a department.

## Competing interests

Dr. L. Cozzi acts as Scientific Advisor to Varian Medical Systems and is Head of Research and Technological Development to Oncology Institute of Southern Switzerland, IOSI, Bellinzona.

## Authors’ contribution

UA and LC designed the study and the analysis. ZO, AA, BG, NK, MY, SP, MS, SG, Generated the plans and collected the data. LC, ZO performed main data analysis. UA and LC drafted the manuscript. All authors reviewed and approved the final manuscript.
